# Applying the dynamic sustainability framework to evaluate the implementation of Cyber-Seniors in higher education: a qualitative interview study

**DOI:** 10.1093/geroni/igaf123

**Published:** 2025-11-03

**Authors:** Rachel M Scrivano, Jill J Juris, Meaghan Colvin, Josie Santilli, Shannon E Jarrott, Skye N Leedahl

**Affiliations:** School of Social Work, Boston College, Chestnut Hill, Massachusetts, United States; Department of Recreation Management and Physical Education, Appalachian State University, Boone, North Carolina, United States; One Sky Community Services, Portsmouth, New Hampshire, United States; Department of Human Development and Family Science, University of Rhode Island, Kingston, Rhode Island, United States; College of Social Work, The Ohio State University, Columbus, Ohio, United States; Department of Human Development and Family Science, University of Rhode Island, Kingston, Rhode Island, United States

**Keywords:** Intergenerational, Technology program, University, Implementation science, Sustainability

## Abstract

**Background and Objectives:**

Technology adoption occurs slower for older adults than other age groups. This consequently reduces opportunities for older adults to stay socially engaged and connected to community resources. Cyber-Seniors (CS) is an intergenerational technology program developed to increase older adults’ digital adoption and competence through reverse mentoring provided by trained students. High school students frequently participate, but higher education is an opportune setting in which mutual benefits can be experienced, including greater positive attitudes toward aging. Despite CS’s successes, research has yet to systematically assess its implementation within higher education. The objective of this study was to explore the determinants of sustainability associated with instructors’ implementation of the CS program in higher education.

**Research Design and Methods:**

This article presents results from the first step of a multi-stage qualitative research study. Semi-structured interviews guided by the Dynamic Sustainability Framework were conducted between April and October 2023. Partnered colleges (*n *= 10) and key CS representatives (*n *= 2) were interviewed over Zoom. Two coders analyzed the data using directed qualitative content analysis in Atlas.ti.

**Results:**

Three themes characterized higher education and CS representative experiences implementing CS: adapting the intervention for higher education, navigating the role of resources, and negotiating external factors within higher education and communities. CS implementation was uniquely challenged due to restrictions related to the COVID-19 pandemic.

**Discussion and Implications:**

This is the first study to apply an implementation science framework to evaluate CS implementation within higher education. Individual programs are tailored to fit their specific practice setting, and several implementation barriers challenge the sustainability of programming. Results suggest that program delivery could benefit from a developed checklist of practices to improve CS implementation within higher education.

Innovation and Translational Significance: Reverse mentoring has been used to increase digital competencies among older adults who are generally slower at adopting new technologies. Research has yet to study the implementation of Cyber-Senior, an intergenerational technology program that has been adopted in higher education settings where students serve as digital mentors, in this context. This study utilizes an implementation science framework to explore determinants of sustainability in CS programs implemented in higher education. Findings can inform educational resources to support the program’s implementation and sustainability in higher education settings to increase the benefits and reach of the program for older adults and college students.

## Background and objectives

Cyber-Seniors (CS) is a non-profit organization that provides older adults with technology training using an intergenerational reverse mentoring model with younger persons serving as digital mentors through CS programs and assists with other programs. Following the lauded CS Documentary (Rusnack & Cassaday, 2014), the organization’s services have evolved. CS began by providing resources to help entities launch their own CS program (e.g., training and recruitment); now, CS offers customizable services to organizations implementing CS programs and to older individuals interested in learning about and engaging with technology.

CS began by offering programs that engage high school students as digital mentors (Rusnack & Cassaday, 2014), but CS can also be implemented within higher education settings. In fact, all academic research on the CS model has been conducted within higher education, including universities, colleges, and community colleges (e.g., Leedahl et al., 2023; LoBuono et al., 2019). Yet, no curriculum or guidelines specific to higher education instructors are available to implement intergenerational technology programs like CS.

CS expanded its offerings in response to the COVID-19 pandemic and heightened understanding of older adults’ need for digital inclusion (Xie et al., 2021). For example, a community organization serving older adults could receive CS support to start its own technology training program or share CS training with older adults. An older adult could receive a one-on-one phone call with a CS technology mentor or attend a CS group webinar on topics like photo editing.

The CS organization regularly conducts program evaluation research through surveys with older participants and/or students; reports on the organization’s website document positive feedback for the program. Reports reflect improved social well-being and increased technology skills for older adults. Younger persons achieve professional skill-building and improved attitudes toward older adults (Cyber-Seniors, 2021). Leedahl and colleagues (2019) were early adopters of CS and have published several articles that have evaluated their higher education model, which mostly involved in-person intergenerational pairs (Breck et al., 2018; Jarrott et al., 2022; Leedahl et al., 2023, 2024; LoBuono et al., 2019). These publications explained the model, adaptations responding to the COVID-19 pandemic, and older adult and student outputs and outcomes. To our knowledge, Juris et al. (2022) are the only other research group that has published results on CS programming within higher education; they described a virtual program that benefited older and young adult participants. The current study builds upon extant literature and fills a gap regarding how intergenerational technology programs utilizing the CS model have been implemented within North American institutions of higher education, which can be hereafter referred to as colleges.

### Implementation science background and use within gerontology research

Because implementation science was born from the movement to improve evidence-based medicine (Boulton et al., 2020), many definitions of implementation science focus on the adoption and integration of an innovation (i.e., intervention, policy, evidence-based practice) within healthcare and public health (Bauer & Kirchner, 2020). In 2006, Eccles and Mittman defined implementation research as “the scientific study of methods to promote the systematic uptake of research findings and other evidence-based practices into routine practice, and, hence, to improve the quality and effectiveness of health services” (p. 1). Since then, Peters et al. (2014) broadened the definition, stating that, “Implementation research is the scientific inquiry into questions concerning implementation—the act of carrying an intention into effect, which in health research can be policies, programmes, or individual practices (collectively called interventions)” (p. 731).

Though most implementation science research is still conducted within health services (Proctor et al., 2023), gerontologists have increasingly been adopting implementation science methods (Bobitt & Jarrott, 2023; Proctor et al., 2023; Sullivan et al., 2023). However, a citation review (Sullivan et al., 2023) revealed that hardly any United States aging studies used implementation science theories, models, or frameworks (TMFs) that focus on sustainability. This is not surprising, knowing that a recent scoping review found that sustainability was one of the least studied implementation outcomes in the field (Proctor et al., 2023). Though sustainability has been diversely conceptualized (Moore et al., 2017), Proctor and colleagues’ implementation outcome taxonomy (2011) defined sustainability as “the extent to which a newly implemented treatment is maintained or institutionalized within a service setting’s ongoing, stable operations’’ (p. 70). Scholars have urged implementation scientists to address sustainability (Estabrooks & Glasgow, 2023; Proctor et al., 2023; Sullivan et al., 2023) and implementation within the post-implementation phase (Sullivan et al., 2023).

Implementation science researchers draw from a plethora (143 and counting; Wang et al., 2023) of TMFs (Strifler et al., 2018). Nilsen (2015) categorized TMFs used in implementation science into five categories: process models, determinant frameworks, classic theories, implementation theories, and evaluation frameworks. Process models can be used to “describe and/or guide the process of translating research into practice” (Nilsen, 2015, p. 3). The Dynamic Sustainability Framework (DSF; Chambers et al., 2013) is a process model developed to increase the chances of an intervention being sustained through continuous evaluation and enhancements to the intervention. DSF relies on the fundamental concept of inevitable change within its key elements: the intervention, the practice setting, and the ecological system. The DSF focuses on increasing fit between these elements through continuous quality improvement. Although scholars have applied the DSF to assess facilitators and barriers to sustainability (Branch-Elliman et al., 2022; Glass et al., 2023; Swindle et al., 2021), few use it to study sustainment (Birken et al., 2020). Due to the DSF’s ability to acknowledge dynamism and change over time, research suggests increasing the use of the DSF to evaluate interventions (Walugembe et al., 2019).

To our knowledge, this is the first study to shift focus from outcomes to implementation, which is essential to facilitate systematic uptake of CS by other potential adopters. This article describes a novel approach to utilizing the DSF to understand the determinants of sustainability associated with implementing CS within North American colleges. The study employed qualitative methods to answer the following research question: What are the determinants of CS sustainability within higher education? The results will advance understanding and improve implementation and sustainability of the CS program involving colleges. Identifying changes that can be made to the CS program and its delivery can help improve CS’s ability to provide expected benefits to students and older adult participants.

## Research design and methods

This directed qualitative content analysis study (Hsieh & Shannon, 2005) was conducted to explore determinants of sustainability associated with the implementation of CS programs within higher education. Data collected through semi-structured qualitative interviews with higher education (university, college, and community college) and CS representatives (i.e., CS organizational staff) addressed their experiences implementing CS. The DSF informed the development of our interview guides and an a priori codebook used for directed qualitative content analysis. This study was approved by the Institutional Review Board (IRB) at Appalachian State University (HS-23-191). Ethical risks for participation in this study were deemed minimal as the interview guides did not include the collection of any sensitive or health-related information. The Standards for Reporting Qualitative Research (O’Brien et al., 2014) guided qualitative reporting.

### Sample

Purposive sampling was used to recruit study participants. Higher education representatives were eligible to participate if they were currently or had previously implemented a CS program among undergraduate and/or graduate students and older adults using any format (e.g., in-person or virtual). Members of the research team were eligible to participate (Probst, 2016) given the small number of CS programs delivered in higher education (Buys et al., 2022). To reduce researcher bias given the dual role of researcher and participant within this study, the following precautions were taken: interviewed research team members (1) did not lead the development of the interview guides and codebook, as this was done by the first author (R.S.), (2) were interviewed first before other higher education representatives, (3) did not interview other higher education representatives as this was led by the third and fourth authors (M.C. and J.S.), (4) led data collection processes and qualitative analysis training (J.J.) but coding and analysis were led by the third and fourth authors, (5) were predominantly involved in later stages of the coding process, including reviewing and suggesting edits to identified themes, (6) used reflexivity when they were involved in the analysis, and (7) did not lead the selection of representative quotes associated with qualitative data as this was done by the third and fourth authors. To understand the role of CS in supporting implementation in higher education that impacts determinants of sustainability, it was also essential to include CS staff perspectives. CS representatives were CS organizational staff with working knowledge of the CS program and how the CS organization partners with colleges.

Participant recruitment was conducted in three phases. First, we contacted 14 colleges that had partnered with CS and met the inclusion criteria. Second, we used posts to share information about the study on professional discussion boards (e.g., relevant Gerontology Society of America’s interest groups, including Technology in Aging and Intergenerational Learning, Research, and Community Engagement). Finally, two CS leaders out of five who were familiar with implementing CS programming were recruited to share their experiences working with higher education partners.

### Interview guide development

The DSF (Chambers et al., 2013) informed the first author’s (R.S.) development of the interview guides (see online supplementary material). This implementation science framework is appropriate because of its emphasis on intervention fit to understand determinants of program sustainability. We conceptualized the DSF elements as follows: the *intervention* as CS, the *practice setting* as higher education, and the *ecological system* as higher education within communities. The interview guides focused on the three DSF elements. Higher education representative questions centered on the CS planning process, implementation, influential practice setting, and ecological system factors, and program evaluation. The CS representative questions focused on CS’s role in program implementation, the extent of CS involvement, and experience within the practice setting and ecological system.

### Qualitative data collection and procedures

Potential participants completed a Google Form to schedule an interview and provide demographic information. A research team member confirmed the interview with a Zoom link and provided an informed consent form to be reviewed before the interview. A confirmation email was sent the morning of the interview. Interviews took place between April and October 2023 and were recorded, transcribed, de-identified, and stored in a secure online platform. All representatives provided verbal consent to participate and have their interview recorded.

Interviews were conducted in English by trained research assistants (M.C. or J.S.) and lasted approximately 60–90 min. Interviewed members of the research team did not interview any college representative participants to reduce researcher bias. Authors three and four (M.C. and J.S.) interviewed college representatives, and authors two and six (J.J. and S.L.) interviewed CS representatives. Interviewees were emailed a link to receive a $99 Amazon e-gift card. Once interviews were complete, a final email was sent to representatives for member checking in January 2024. In this email, the research team shared the patterned codes to summarize key points for intervention, practice setting, and ecological system. Representatives could provide input and ask questions to ensure the findings accurately captured their experience implementing CS. Member checking allowed for triangulation (Padgett, 2008) as we shared concepts identified through analysis with participants to allow for confirmation or edits. Six of the 10 higher education representatives responded during the member checking process, indicating overall agreement with the findings. Based on emailed instructions, participants’ non-responses also indicated agreement with the findings.

### Qualitative data analysis

After receiving initial training by the second author (J.J.), two coders (M.C. and J.S.) led analysis using a directed qualitative content analysis approach (Hsieh & Shannon, 2005). A codebook was developed (by R.S.) as a data management tool (Crabtree & Miller, 1999). A priori codes were created (by R.S.) based on components of the DSF (Chambers et al., 2013). The coders then reviewed two transcripts to test the codebook, created additional subcodes as necessary, and refined code definitions (Crabtree & Miller, 1999). Analytic memos were used to then code the transcripts segment-by-segment (Saldaña, 2021) to integrate reflexivity. To move analysis toward primary themes, based on the codes, the coders created a list of descriptive themes (Shaw, 2019) and a visual thematic map (Braun & Clarke, 2006). Code modifications and memos were maintained throughout the analysis process in Atlas.ti. Coders shared themes and patterns with the research team through regular meetings and discussions until consensus was achieved. Finally, transcripts were reviewed for disconfirming evidence that is contrary to the pattern codes. Coding occurred throughout data collection. Saturation was achieved (Padgett, 2008) after meeting with 10 higher education representatives and two CS representatives, as no new information was heard, and participant responses were being repeated.

## Results

### Participant characteristics

Ten higher education representatives, including two authors of this article, and two CS representatives, were interviewed. All higher education and CS representatives were women. The two CS representatives held staff positions that were most closely connected with implementing CS programming in college settings, with their roles being a mentor program director and a project manager at the time of the interview. The average age of participants was 47 years (*SD *= 12.49, range = 20–69). Most representatives were White (70%), with 20% identifying as Asian and 10% identifying as Black/African American, American Indian, or Alaska Native. Most identified as Non-Hispanic or Non-Latino (90%). Participants had a range of 2–20 years of employment at their colleges, with an average of 10.10 years.

### CS project characteristics

Colleges were located across North America, including the United States and Canada, with most representatives located in urban areas (70%), followed by rural areas (30%). Representatives reported a variety of delivery platforms for CS programming, including fully online (*n *= 5), in-person only (*n *= 2), and hybrid (*n *= 3). Four out of the 10 CS programs were active at the time of the interview; these programs provided either continuous programming before, during, and after the COVID-19 pandemic (representatives 02 and 06) or beginning during and continuing after the pandemic (representatives 04 and 07). Four (representatives 01, 03, 08, and 09) programs only operated during COVID-19, and two programs (representatives 05 and 10) started after the pandemic.

Three themes (see Table 1) aligned with the DSF elements that characterized higher education and CS representatives’ experience implementing CS programming: (a) adapting the intervention for higher education (intervention), (b) navigating the role of resources (practice setting), and (c) negotiating external factors within higher education and communities (ecological system). Three to four codes were included within each theme (see Table 1).

**Table 1.** Themes, codes, and operational definitions.

Theme	Codes	Operational definitions
**1: Adapting the intervention for higher education**	Students as technology mentors	Adapting how students engaged in the university program (e.g., service-learning, volunteering, class credit, internship, paid work-study position).
	Delivery format	Adapting how the program is delivered to participants (i.e., online, face-to-face, frequency).
	Training	Adapting teaching a student or university representative the required skills and behaviors necessary for program participation and onboarding participants.
**2: Navigating the role of resources**	Recruiting	The method by which older adults and university students are informed of and enrolled in the program.
	Balancing program needs with Cyber-Seniors support	The extent to which the Cyber-Seniors organization assisted university program leaders. The presence and ability of university program leaders (i.e., graduate students, paid staff, faculty) to assist student mentors, schedule sessions, and provide program oversight.
	Funding	Amount of financial resources available to support program costs.
**3: Negotiating external factors within higher education and communities**	Partnerships	The connection between stakeholders who share a vested interest in the sustainable operation of the program.
	Campus culture	A common set of beliefs and ideas that determine the social norms of the university setting.
	Policies and regulations	Procedures and requirements for programming and research to occur.
	Macro forces	Major external events impacting programming (economic, demographic, political, etc.).

### Adapting the intervention for higher education

Reflecting the intervention element of the DSF, higher education representatives needed to adapt the CS intervention to fit their college. They described adaptations related to students as technology mentors, delivery format, and training.

#### Students as technology mentors

Each higher education representative considered how to engage college students in their college’s CS program (e.g., course credit, wage pay, or unpaid volunteer). Of four active programs, two engaged students in 10–40 hr for credit, one paid a student, and one conducted research. The six inactive programs involved students as volunteers, who earned neither course credit nor pay.

College representatives who integrated CS into course requirements identified course barriers related to balancing their college’s service-learning course requirements, students’ other commitments, transportation, and older adults’ interests. The college representative (01) echoed this challenge, noting the importance for instructors to balance coursework and the CS hours: “making sure that you are allocating time you have your students with you.” Difficulties building a robust mentor base related to challenges relying on students, “to commit on any ongoing basis,” (representative 06). Particular times during the academic terms were more conducive to student engagement than others. For example, a higher education representative (03) explained, “We did find that there were certain times of the year that were a little easier to recruit young people because that was their window for when they wanted to do service. So later in the school year wasn’t necessarily the best way to go.”

While locating in-person sessions off campus worked best for older participants, students experienced barriers related to expenses, time constraints, and transportation access. Student attendance was easier to secure for CS programs that were near older adults. A CS representative (01) suggested that colleges offering in-person sessions “need to at least provide stipends for travel, at the very least, especially if you’re asking [students] to go somewhere.”

#### Delivery format

Issues related to delivery format included the method of delivery (i.e., online, hybrid, or in-person) and factors that influenced how students and older adults were paired. Considering the delivery format of the active programs, two were hybrid, one was delivered online, and one met face to face. Four of the six discontinued programs had been online, one was face to face, and one was a hybrid of online and face to face. While an online platform, such as Zoom, proved helpful during the COVID-19 pandemic, representatives emphasized that older adults prefer in-person contact, whether using an in-person or hybrid intervention format. As representative 05 explained, “They [older adults] really do not want to be referred to videos. And they do not want to talk to bots on the phone. They want to sit down with someone face-to-face and learn.”

Program frequency impacted whether students and older adults were paired. Five programs (three active and two inactive) met multiple times. Higher education representatives used varied approaches to match technology mentors and older adults, including aligning student skills with older adult interests. Requiring attendance at a scheduled session or allowing students and older adults to schedule their own appointments also enabled consistent pairing across sessions. Repeated contact allowed technology mentors to establish trusting relationships with older adult participants, contributing to positive outcomes. As representative 06 explained, “many of them had some really great experiences with some one-to-one mentoring in particular, and some had the opportunity to mentor the same person for multiple sessions, making some of those connections.”

Some programs held one-time meetings online, which helped, “people who just have a quick question, ‘My printer’s just not connecting,’ otherwise they don’t need that much help,” (CS representative 01). In these circumstances, pairs met once, based on the older adult’s technology interest and the student’s skills.

#### Training

Another element of adapting the CS intervention for higher education involved training students and faculty. The CS organization offered two training options. Colleges can conduct training on their own or utilize CS training modules. The former might involve an in-person or synchronous online training where the students sometimes also watch the CS documentary, while the latter involves students completing six self-paced training modules and quizzes before initiating technology mentoring. Those who utilize both could do an in-person training and ask the students to complete the CS modules. Eight of the 10 programs utilized training modules provided by CS; two programs did not use the training modules. Six of the 10 programs integrated the CS documentary into preparing students for participation. College representative 07 articulated:

…the purpose of [students] getting the training isn’t so much to teach them technology or how to use the technology, but how to work effectively with older adults, how to be patient, how to maintain their calm when working with older adults, speaking clearly and loud enough so an older adult can hear them, those kinds of things.

Higher education representatives benefited from the CS training materials, which provided consistency and established a knowledge base. Training students to use older-adult-specific communication skills and be patient helped them feel more prepared to provide technology support to older adult CS participants.

Besides training materials, the CS organization offered an online platform for students to schedule and host CS meetings with older adult participants and track their hours. Higher education representatives expressed a sense of unpreparedness to onboard students to the CS platforms. As college representative 09 recalled:

A lot of [students] did find it really confusing. I remember there was a lot of coming back and asking, “Okay, now we sign up here, then we sign up here, how does this work? How does that work? This password doesn’t work for that.”

The CS representatives agreed that platforms for scheduling and online meetings were perceived as barriers for some programs.

### Navigating the role of resources

The theme of navigating the role of resources aligns with the DSF element practice setting, with resources identified as an essential component to implement CS in higher education. Practice setting characteristics encompassed older adult and student recruitment, the involvement of CS at each college, and funding.

#### Recruiting

Older adults are a crucial resource for program implementation in the practice setting. Higher education representatives relying on in-person interactions emphasized the importance of forming alliances with local institutions (e.g., senior centers, Osher Lifelong Learning Institutes) to ensure a steady flow of older adult participants for program sustainability. Colleges lacking such alliances or the resources to establish them, along with those adopting an online delivery format, could delegate recruitment of older adults for online programming to CS. When asked about recruitment, one higher education representative simply replied, “We don’t have to do that, Cyber-Seniors does that” (representative 06). Another higher education representative explained, “…when you don’t have the capacity to run a program, you can effectively run [the online] program through Cyber-Seniors with very little administrative challenges” (representative 03). Similarly, a CS representative (01) acknowledged their value as recruiters: “The virtual component for schools is quite popular and quite good [for some colleges]. It also takes a lot of pressure off the teachers because they’re not looking for the seniors.”

As for recruiting student technology mentors, programs that struggled to recruit student volunteers agreed that providing a stipend might be a more viable option. As college representative 05 explained:

It’s been really challenging to get volunteers to commit on any ongoing basis. So, I’ve temporarily revamped the program and I’ve been paying my work study student to serve in this role…because I was finding that the young folks who were just volunteering were often not showing up or not getting back to me. So, I think one of the challenges of the program is that it relies on volunteers.

#### Balancing program needs with CS support

Desire for interaction with the CS organization varied across higher education representatives. Leaders of in-person programs that intended to foster intergenerational relationships were more likely to incorporate a research component. These leaders preferred limited interaction with CS. In contrast, programs focused on students gaining service-learning experience relied more on CS, such as recruiting older adults.

For programs operating with an in-person component (*n *= 5), higher education representatives indicated a heavy reliance on human resources, such as graduate student staff, collegial faculty/staff, and community partners. As representative 02 stated, “I’ve been able to add some doctoral students and more qualified master’s students who have been really pivotal in helping maintain [the program].” Facilitators to program implementation in higher education included employing graduate students and having faculty or program leaders supervise student participants. Challenges arose when students were under 18 years of age, necessitating additional support for program facilitation.

A notable challenge of initiating an in-person or hybrid program in higher education was the administrative workload. Most higher education CS programs operated with one to five higher education administrative staff members. Having a dedicated team with adequate allotted time contributed to effective program operations. For some, building and managing this team proved challenging, as described by representative 02:

It is very tricky, monitoring the needs for all of these different folks. I’m on my email constantly. I respond to emails all day, and I try to respond to my community partners and my students very quickly. It’s just been key to doing this, but it gets to be a lot sometimes.

#### Funding

Most programs (*n *= 8) had at least some external funding support. Access to grant funding and resources, including technological devices, enhanced intervention delivery, and the ability to collect data. For example, “The thing that made it possible to do such robust surveys with the older adults has been the funding and the iPad [given to older adult participants as an incentive]. That’s substantive enough that they are willing to do our surveys” (representative 02). Funding enabled programs to hire staff to assist with the program, thereby reducing turnover associated with student employees. Additionally, funding sometimes supported marketing efforts and high-quality materials, incentives, and the provision of the Internet, devices, and/or other technology support services.

### Negotiating external factors within higher education and communities

The final theme, negotiating external factors within higher education and communities, incorporates the following elements in relation to the ecological system of the DSF: partnerships, campus culture, regulations/policies, and macro forces.

#### Partnerships

Higher education and CS representatives commented on the importance of community partnerships. Three of the four active programs had partnerships with local organizations serving older adults (i.e., senior center, senior living community). Another partnership involved Cooperative Extension. CS representatives shared observations regarding these partnerships. One CS representative (01) stated, “You have to find where your seniors are, and you have to find where your students are. Typically, [higher education partners] have one of those two things. It’s very rare that we find that the person was coming to us with neither group already established.” The CS organization attempts to guide colleges in creating their own CS programs, expecting that local instructors and administrators are best positioned to build community alliances. As one CS leader (01) said, “We always suggest partnering with a local library that has a strong senior pull.” When CS programming involves in-person or hybrid formats, local connections prove essential and cannot be outsourced to the CS organization.

#### Campus culture

The campus culture also contributed to program implementation. When department leadership and higher education administrators embraced the partnership with CS as a source for service-learning for students, grant applications and mentor recruitment were supported, thus contributing to program success. Others discussed that, “there was some dynamic of not feeling fully supported in figuring it out… [higher education administrators] want us to bring in grant funding and don’t really want to help with the day-to-day [requirements]” (representative 01). Establishing partnerships with faculty members was a barrier for several higher education representatives (*n *= 4). Respondents noted that some faculty declined to participate because of concerns that the time required for CS would negatively affect faculty performance and promotion reviews, such as student evaluations and time for other duties.

#### Policies and regulations

Policies impacting in-person CS models played a crucial role in some programs (*n *= 4). One interviewee described care settings like retirement homes, a promising source of older adult participants, as “hard to get into [with] strict laws…going beyond a background check [limiting student access]” (representative 02). An additional regulatory barrier experienced by representatives (*n *= 3) consisted of research and human subject protections required for student interactions with older adults. State-specific regulations and training requirements created another layer of policies to navigate. One respondent stated, “… in addition to IRB, we have to follow protocol by [Department of Aging] or by the state when conducting this research” (representative 04). Some colleges (*n *= 3) specifically avoided utilizing students under 18 years of age to avoid regulatory barriers.

#### Macro forces

Finally, the COVID-19 pandemic was a concern for implementing CS programming. In-person programs had to adapt quickly or halt programming due to safety regulations imposed by higher education and older adult settings. Adaptations limited the opportunity for interaction (i.e., meeting online, meeting outside). The COVID-19 pandemic illuminated the need for technological competence in older adults as most social and professional commitments shifted to an online delivery format such as Zoom and Webex. CS programs met this need by providing webinars on needed competencies to join programs on various platforms.

## Discussion and implications

CS is an intergenerational reverse mentoring technology program by which younger volunteers provide older adults with technological training. While high school students commonly participate in CS programming, available research on CS has solely focused on social and skill-related outcomes experienced by older adults and college student participants (Breck et al., 2018; Cyber-Seniors, 2021; Jarrott et al., 2022; Juris et al., 2022; Leedahl et al., 2019, 2023, 2024; LoBuono et al., 2019). Despite evidence pointing toward program benefits, research has yet to evaluate the implementation of CS in higher education. Identifying determinants of CS sustainability not only addresses a gap within IS (Proctor et al., 2023) and aging research (Sullivan et al., 2023) but also provides needed evidence for researchers to develop resources to aid the implementation of CS in higher education. This was the first study to explore determinants of sustainability associated with CS program implementation in higher education.

### Key findings

Three themes were generated through a directed qualitative content analysis approach (Hsieh & Shannon, 2005): adapting the intervention for higher education, navigating the role of resources, and negotiating external factors within higher education and communities. These three themes aligned directly with the three elements of the DSF. The first theme of adapting the intervention for higher education aligns with the DSF’s element of intervention, the second theme of navigating resources aligns with the practice setting element, and the final theme around the importance of negotiating external factors aligns with the framework’s element of the ecological system (Chambers et al., 2013). Together, these themes illustrated the importance of enhancing the fit of an intervention within multi-level contexts of the practice setting and ecological system (i.e., the CS organization and other entities and policies within higher education) for supporting sustainable implementation (Chambers et al., 2013). While other educational intergenerational interventions focus on conversation, shared movement (e.g., physical activity), or engagement with physical materials like games, CS’s technology component places additional demands on the practice setting (e.g., electricity, internet connectivity, equipment functionality), thereby adding another dimension to account for in program and participant preparation.

These themes align with several intergenerational best practices that were previously identified in a scoping review that primarily included university students as participants (Jarrott et al., 2021b). For example, the themes of adaptation and resources, respectively speak to the need to be flexible with structured activities, set/select the environment, and prepare staff and participants. Our third theme of negotiating external factors mirrors an intergenerational concept of program pivoting, especially when macro forces like the COVID-19 pandemic limit opportunities for participants to engage together in person (Jarrott et al., 2022). Findings also extend Roodin and colleagues’ (2013) suggestions for future directions in intergenerational service-learning related to assessing community costs and benefits that fit within negotiating external factors within higher education and communities. This study also identified the need for internal and external support for programming to increase buy-in. Community-based participatory approaches complement these results and can feasibly be used to support intergenerational programming that involves community partners and participants (Jarrott & Lee 2019; Jarrott et al. 2021a).

When implementing CS programming in higher education, instructors should consider additional factors that may not have been important or relevant for CS implementation in other contexts (e.g., high school). Instructors should plan how CS programming will be integrated within higher education. Integration should allow for optimal student participation (e.g., providing program opportunities through courses such as service-learning; Leedahl et al., 2019) where students will be motivated, have time, and have access to resources (e.g., space, transportation, training materials, technology). Compensation for participation outside of benefits from volunteering should be considered, as students may need to rely on their own finances to participate or give up time they otherwise could have used toward a paid position. However, this may not always be possible. Delivery of the intervention should also cater to preferences older adults may have, such as the mode of delivery (i.e., in-person or online) to ensure the program is compatible and useful for both age groups (Jarrott & Lee 2019), increasing its chances of being acceptable and appropriate (Proctor et al., 2011). While our results indicated that colleges utilized different program models (e.g., whether the CS organization was hands-on or hands-off with regard to providing and facilitating pre-implementation activities such as training, recruitment, and ongoing support), college instructors should consider leveraging existing resources from the CS organization and/or those accessible from their home college to support program sustainability. The degree to which a college involves the CS organization in programming may depend on funding, staffing, time, and community connections. For example, colleges have to pay to partner with and utilize CS organization resources. Colleges that have long-standing partnerships with local community centers (e.g., senior center) may not need to involve the CS organization as heavily. Regardless of one’s available resources, results indicated that implementing a CS program is time-consuming and requires a team to organize and oversee its implementation. Accessing support from willing and available sources to implement evidence-based programs is not unfamiliar to intergenerational work (Juris et al., 2024).

Based on the nature of this work, intergenerational programs require additional considerations. Unlike other educational interventions, CS relies on using technology to foster intergenerational connections (Leedahl et al., 2019). Intergenerational contact in combination with positive educational experiences can provide mutual benefits to both students and older adult participants, as supported by the positive education about aging and contact experience model (Levy, 2018). While policies and regulations that are put in place to protect older adult participants are important and necessary, they can introduce implementation barriers for college instructors and students (e.g., vaccinations and paperwork completion). Understanding how to navigate these potential barriers early, prior to CS implementation, can improve program implementation and outcomes for participating groups. Relatedly, many of these programs also encountered implementation challenges due to changing policies and regulations around the COVID-19 pandemic. It is essential to have a plan to appropriately pivot programming when outside factors impact program implementation to ensure program participants receive support and benefits through continuously delivered programming (Jarrott et al., 2022). For example, the pandemic caused intergenerational programs to shift from in person programming to a virtual format. Implementers had to be cognizant of participant skills, technology accessibility, and available resources when deciding how participants could continue contact (e.g., selecting Zoom or phone calls). Other projects had to consider new participant needs and adjust programming to address these project-related matters (see Jarrott et al., 2022 for more examples).

### Implications and future directions

This is the first study to provide initial evidence of determinants related to sustainable implementation of an intergenerational technology program within higher education. While past research has evaluated evidence-based practices used in intergenerational programming (Jarrott & Lee 2019, Jarrott et al. 2021b, 2022), research has yet to develop and test implementation strategies (i.e., interventions that support the implementation of an innovation) to address implementation barriers and leverage facilitators for intergenerational reverse mentoring programs in higher education. The discrete (i.e., singular) implementation strategy of developing educational materials (Powell et al., 2015) may be appropriate for supporting the sustainable implementation of the CS program in higher education, as there currently is no resource guide or formal program manual that is accessible to college instructors. The educational material can provide information about the CS program and practices that can aid the implementation and delivery of the program within higher education, based on the type of resources and level of support the instructor has available to them.

Practices should be created with sustainability in mind. While financial strategies are one of the most commonly reported sustainability strategies for evidence-based interventions (Hailemariam et al., 2019), it may not be appropriate for most instructors who do not have funds to partner with CS for program delivery activities, provide stipends to program participants, and provide funding support to team members. Based on our results, practices may center around readily available resources. For example, practices may include recruitment strategies based on instructors’ available funds and community partnerships. While it is important to support the implementation of CS in higher education, other intergenerational technology programs exist (e.g., Tech Pals and Conversations to Remember). Educational materials should be made to generalize to other intergenerational technology programs to benefit other program types. Future research should systematically evaluate the effectiveness of these practices and assess their impact on implementation sustainability.

### Limitations and strengths

This study has several limitations. First, we learned that there were few programs that have implemented CS in higher education. Therefore, our potential sample size was limited and included two members of the research team. While our team took several precautions to reduce the risk of bias in this study, the presence of the dual role of research and participant still presents potential bias. This may particularly be the case in that the interviewed research members did review and provide feedback on the developed interview guides initially drafted by the first author (R.S.), one of the interviewed research members led the training for our two coders, and both interviewed research members reviewed and discussed initial identified themes. It is important to note, however, that responses of higher education participants who were part of the research team did not, to our knowledge, differ in any noticeable ways from other participants. Despite our small sample size, our results may be able to generalize to other intergenerational technology programs implemented in higher education as well as other CS programs. Developing educational materials can advance the CS program so others can readily implement it within their college. Second, we did not compare the implementation of CS outside of higher education. Doing so would have highlighted determinants that are generalizable to other contexts and specific to others. Last, there was a lack of diverse representation among representative colleges interviewed; many representatives were White and were associated with urban colleges that may have greater access to resources as compared to colleges in rural settings. Understanding the implementation of intergenerational technology programs in other higher educational contexts may highlight both reach of the program and potentially unique determinants.

Our study has several strengths. This was the first study to assess determinants associated with implementation sustainability for intergenerational programs implemented in higher education. The DSF (Chambers et al., 2013) was thoroughly used to guide data collection, analyses, and interpretation to enhance the rigor of our work. Results of this study help to better understand factors that impact implementation sustainability within higher education, an understudied topic in both implementation science (Proctor et al., 2023) and aging (Sullivan et al., 2023). Along with member checking, we were also able to incorporate perspectives from two CS representatives to triangulate, compare, and contrast higher education representative perspectives. This inclusion enhanced our results as we were better able to more completely understand determinants related to the ecological system of the DSF (Chambers et al., 2013).

## Conclusion

This was the first study to investigate determinants of implementation sustainability for the implementation of the CS program in higher education. Our results aligned well with the DSF framework and highlighted several factors related to implementation sustainability pertaining to the intervention, resources, and external factors. Results from this study can be used to inform implementation strategies to address barriers to sustainability and enhance program implementation and delivery.

## Supplementary Material

igaf123_Supplementary_Data

## Data Availability

Analytic methods and materials are provided in this article (i.e., see methods section and supplemental material), but the data supporting this study are not publicly available due to ethical restrictions involving participant confidentiality and privacy. This manuscript was not preregistered.
