# Supplementary material for: Applying the dynamic sustainability framework to evaluate the implementation of Cyber-Seniors in higher education: a qualitative interview study
Source: Innov Aging. 2025 Nov 3;9(12):igaf123. doi: 10.1093/geroni/igaf123 (PMC12711305; doi:10.1093/geroni/igaf123)
Supplement: igaf123_Supplementary_Data [file igaf123_supplementary_data.docx]

***Innovation in Aging* Supplementary Material: Scrivano et al. Applying the Dynamic Sustainability Framework to Evaluate the Implementation of Cyber-Seniors in Higher Education: A Qualitative Interview Study.**

**Interview Guide for Higher Education Representatives:**

Thank you for taking the time to meet today to discuss your experience with implementing Cyber-Seniors. We provided an email with the full informed consent form, including an overview of the project, risks, and benefits.

Do you have any questions?

This interview should last approximately 60 - 90 minutes and we would like to record it to transcribe your responses for analysis. We will transcribe your responses and assign you a unique number code. Individual records will be labeled with this code and only authorized study staff are allowed access to the code key.

Do you consent to participating and being recorded?

If they say no: Thank you for your time. Have a nice day.

If they say yes, proceed:

This project is funded by the RRF Foundation for Aging, and the researchers are not employed by Cyber-Seniors. The goal of this first step of our study is to understand factors and practices that impact the implementation of Cyber-Seniors in higher education. We would like you to share what has and has not worked for your program throughout the duration of your program.

Upon completion of the interview, you will receive an Amazon $99 e-gift card that will be provided to you through the University of Rhode Island.

We are going to talk about three topics today: the Cyber-Seniors program, implementing the program, and your university.

**To start, we would like some background information about your program.**

1. How long (how many years) has your Cyber-Seniors program been operating?
   - When did your program start?
   - When did your program end?
2. How long does your program and each session last? (e.g. All semester, 6 weeks, summer, etc.)
3. What is the goal or mission of your Cyber-Seniors program?
4. Who are your participants?
   - Who are the students involved? (e.g., course, required/elective, undergrad/grad).
     1. Do students have a background/interest in gerontology?
     2. Do the students have to complete a certain number of hours or meet certain requirements?
   - Who are the older adults involved? (e.g., community members, senior center, retirement community)
     1. Do older adults agree to a specific time commitment?

**Great, thanks. Next, we would like to learn more about your specific program. We will start by asking for a program overview and then the key components of your program. In the *next section* we will focus on the planning process, implementing the program, and finally program evaluation.**

1. Please give an overview of how your program works.
   - What is the format of your program? (i.e. meeting face to face, fully online, hybrid)
   - Where does your program take place? (i.e., in the library on campus, over Zoom)

- How do you approach accessibility and inclusivity? (e.g. Apple vs HP, closed captioning, materials in advance)
- How do you consider the safety of your participants in person and online? (e.g., background checks, online safety-passwords, in-person meeting safety, professionalism, protecting privacy)

1. How would you describe the key elements of your program?
   - How do you pair participants?
   - How do you prepare the environment?
   - How long does each Cyber-Seniors session last? (e.g., one hour, two hours, variable times)

1. Can you please describe your program’s geographic location?
   - How does that influence your program decisions?

**Okay, we are about halfway through our questions, let’s now move into the planning process, actually implementing the program, and your program evaluation.**

1. How would you describe your program’s **planning process**?
   - How do you recruit participants?
   - Do you screen participants (for previous knowledge, technology use)? If so, how?
   - How do you market your program?
   - How do you prepare participants?
   - What platforms do you use?

1. Please walk us through how you **administer and implement** your Cyber-Seniors program, step-by-step.

- Think of when you stopped planning and started implementing, what did that look like?

1. When you think of each step needed to make your program run, what implementation strategies have worked well?
   - What do you believe has been key to your success so far?
   - Others mentioned… Did you encounter similar…
   - You mentioned…. What made that work well

1. What implementation strategies haven’t worked?
   - What barriers have kept things from running smoothly?

1. Who is involved in delivering your program?

1. How do you measure program outcomes for your Cyber-Seniors program?
   - What outcomes are you measuring?
   - Are both students and older adults measured?
   - What measures do you use?
   - How did you choose the measures you use?
   - What methodology do you use? (i.e., qualitative, quantitative, mixed methods)
   - Generally speaking, what are your findings to date?
   - What studies are you planning to do in the future related to Cyber-Seniors?

**We would now like to learn more about your staffing, training and organizational structure and culture.**

1. How are program responsibilities shared?
   - Who is involved in program delivery?
   - How are student leaders involved?
     1. What are their responsibilities?
   - How are day-to-day operations supervised?
   - Do you have adequate staffing?
     1. Are there any aspects of staffing you would change?
   - How do you build comradery among staff?
   - How would you describe your organizational culture?

1. How do you approach training?
   - Who receives training?
   - What are the goals of training?
   - What training and/or resources from the Cyber-Seniors organization do you use?
   - What training materials have you created specific to your program?
     1. What do you hope participants gain from training?
   - What other training or resources would be helpful to you and your participants?

**Okay we are now in our last section of questions, how are you doing with our time? This last section pertains to your university structure and policies.**

1. How do curricular requirements, regulations, and policies within higher education impact the implementation of Cyber-Seniors at your institution?

- What facilitators do these regulations introduce?
- What barriers do these regulations introduce?
- Is accreditation a consideration for you? How?
- What practices would help to mitigate these regulation barriers?

1. How is your program regarded by others at your university/college?
   - Departments?
   - Students?
   - Faculty and staff?
   - Dean?
   - President/Chancellor?
   - Other colleges?
   - How do they support your program?

15. Those are all of our questions. Is there anything else you would like to add about your experience implementing the Cyber-Seniors program at your school?

Thank you for your time today. We really appreciate your insights. We will stop recording now.

**Interview Guide for Cyber-Seniors Staff**

Thank you for taking the time to meet today to discuss the implementation of Cyber-Seniors. We provided an email with the full informed consent form, including an overview of the project, risks, and benefits. Do you have any questions?

This interview should last approximately one hour and we would like to record it to transcribe your responses for analysis. We will transcribe your responses and assign you a unique number code. Individual records will be labeled with a unique number code and only authorized study staff are allowed access to the code key.  Do you consent to participating and being recorded?

If they say no: Thank you for your time. Have a nice day.

If they say yes, proceed:

This project is funded by the RRF Foundation for Aging. The goal of this first step of our study is to understand factors and practices that impact the implementation of Cyber-Seniors in higher education. We would like you to share your knowledge of and administrative experience with the Cyber-Seniors program.

Upon completion of the interview, you will receive an Amazon $99 e-gift card that will be provided to you through the University of Rhode Island.

We are going to talk about two topics today: the Cyber-Seniors program and implementing the program in higher education.

**To start, we would like a general overview of your program.**

1. How long (how many years) has the Cyber-Seniors program been operating?
2. What is the goal or mission of the Cyber-Seniors program?

**Great, thanks. Next, we would like to learn more about your program specifically with how you work with partners in higher education. We will start by asking about barriers and facilitators within the higher education context. In the next section we will focus on the planning process, implementing the program, and finally program evaluation. Finally, we would now like to learn more about your staffing, training and organizational structure and culture.**

1. How does the format of Cyber-Seniors in higher education compare to your other program settings that don’t include higher education? How have you adapted?

**Barriers and Facilitators**

1. Please share your perspective on what implementation strategies have worked well for college and university instructors.
   - What do you believe has been key to their success so far?
   - What could work better?
   - For programs that have been sustainable overtime, what has seemingly worked in these situations?
     1. Recruiting? Class requirements? Internships?
   - Are there aspects of other educational settings (such as high schools, after school programs) that make it easier to implement Cyber-Seniors?
2. Thinking about the college and university setting, what barriers have you come across that make it difficult to implement Cyber-Seniors?
   - What implementation strategies haven’t worked for college and university instructors?
   - What barriers have kept things from running smoothly?
   - Are there aspects of the educational setting that makes it more challenging?

____________________________________________________________________

**In the next section we will focus on the planning process.**

1. To begin, let’s start at the beginning with how Cyber-Seniors works with educational partners (like high schools, colleges, universities, after school programs). When a partner initially reaches out to you to show interest in a partnership, what happens? Please walk me through a typical situation in these early stages.
   - What decisions need to be made?
   - Who from Cyber-Seniors do they usually work with?
   - What types of questions do you ask them?
   - Where do most of your student mentors come from, meaning what type of educational setting?
   - What do you expect university partners will provide/do?
2. When universities/colleges reach out to you, what do they request from you in order to run their program?
   - Are there different requests depending on how the program will be delivered? E.g., face to face, fully online, hybrid, setting differences, age group differences, etc.
   - How do you meet their needs? E.g., provide them with the participant’s handbook, the start-up manual, toolkits, discussion and activity guides, a partner’s registry, a curriculum, outside resources, support, advice, technology assistance, etc.

**Okay, let’s now move into actually implementing the program and your evaluation of the program.**

1. Please walk us through your role in helping an instructor implement Cyber-Seniors programming, step-by-step.
   - How do you know if they are prepared?
   - Who on your team do they meet with next?

**EVALUATION**

1. What outcomes of Cyber-Seniors programming are you most interested in measuring?

**We would now like to learn more about your staffing, training and organizational structure and culture.**

1. How are program responsibilities shared?
   - Who makes up your team?
   - How are day-to-day operations supervised?
   - Do you think your team is adequately staffed?
     1. Are there any aspects of staffing you would change?
   - How do you build comradery between Cyber-Seniors and university partners?
   - How would you describe your organizational culture?
2. What training or support does your team provide to university partners? [skipped-discussed time]

Our last section of questions pertains to university structure and policies.

1. Have you identified any higher education policies, regulations, or curricular requirements that Cyber-Seniors has needed to consider when helping implement the program within higher ed? If so, can you provide some examples?
2. What do you think about higher education as an implementation setting for Cyber-Seniors?
   - Is it sustainable?
   - Why do some programs stop running their programs?

Those are all of our questions. Is there anything else you would like to add about your experience helping to implement the Cyber-Seniors program within colleges and universities?

Thank you for your time today. We really appreciate your insights. We will stop recording now.
